# Human Interferon Alpha-2b: A Therapeutic Protein for Cancer Treatment

**DOI:** 10.1155/2014/970315

**Published:** 2014-03-10

**Authors:** Ratih Asmana Ningrum

**Affiliations:** Research Center for Biotechnology, Cibinong Science Center, Jalan Raya Bogor km 46, Cibinong, Bogor, West Java 16911, Indonesia

## Abstract

Human interferon alpha (hIFN*α*) is a wide biological activity cytokine that is used in hepatitis and cancer treatments. It regulates many genes that are involved in antiviral and antiproliferative activities. This mini review focuses on human interferon alpha-2b (hIFN*α*-2b) as therapeutic protein for cancer treatment. The review covers hIFN*α*-2b molecular characteristic and its molecular mechanism by Janus activated kinase/signal transducer activation of transcription (JAK-STAT) pathway. The JAK-STAT pathway regulates not only proteins involved in inhibition of proliferation but also apoptosis. As additional discussion of clinical applications, the use of recombinant hIFN*α*-2b (rhIFN*α*-2b) as therapeutic protein in several types of cancer is also explained.

## 1. Introduction

Cancer is a first cause of death in developed countries and the second cause of death in developing countries. It was reported that approximately 12.7 million cancer cases occur worldwide with 7.6 million of mortality rate. About 64% of deaths arise in developing countries. Breast cancer in women and lung cancer in men are the biggest cause of death, followed by stomach, liver, cervix, and prostate cancers. Cancer is a disease caused by uncontrolled cell growth. Growth of these cells causes damage to surrounding tissues and spreads to other tissues via the blood or lymphatic circulation [1, 2].

Cancer is generally derived from one cell with accumulated changes in several different genes. Mutation can be caused by exposure to carcinogens, radiation, or infection by bacteria and viruses. Accumulation of mutations causing abnormal cell growth is known as premalignancy. Genes that are involved in cancer are divided into two categories: oncogene and tumor suppressor. Protooncogenes are protein-coding genes that promote cell division, including growth factors (e.g., platelet-derived growth factor, epidermal growth factor, and fibroblast growth factor), growth factor receptors (e.g., HER2 ERBB2, ERBB1, and IGF-1R), and proteins in signal transduction pathways (e.g., RAS, ABL, and RAF). Oncogenes are the mutated forms of protooncogenes. Mutation in oncogenes causes different characteristics or overexpression of protein that triggers the growth of abnormal cells. Tumor suppressor genes are protein-coding genes that suppress cell division, such as RB1 and p53. Mutations in tumor suppressor lead to loss of suppressor activity so that the cells become abnormal in growth. Abnormal cells are used as a therapeutic targets for cancer treatment [2, 3].

Cancer treatment can be performed in various ways, such as surgery, chemotherapy, radiotherapy, immunotherapy, gene therapy, or protein therapy. Recombinant therapeutic proteins that have been widely used in cancer are enzymes (Elspar, Oncaspar, and Elitek), toxins (denileukin diftitox or ontak), monoclonal antibodies (Zevalin, Mylotarg, Bexxar, Herceptin, Avastin, Erbitux, Rituxan, Vectibix, and Campath), and cytokines (interleukin-2, interferon-*α*n3, interferon-*β*1, and interferon-*α*2b). rhIFN*α*-2b was first approved as therapeutic protein by United States Food and Drug Administration (FDA) in 1986. It is used for the treatment of hairy cell leukemia and currently as much as 86 countries have been using rhIFN*α*-2b in hepatitis and cancer treatments [1, 4]. rhIFN*α*-2b is widely applied in monotherapy or in combination therapy with other drugs. rhIFN*α*2b is combined with ribavirin, lamivudine, or adevofir on hepatitis treatment and combined with cytarabin, vinblastine, 5-fluorouracil, tamoxifen, or interleukin-2 on cancer treatment [4–6].

## 2. Molecular Characteristics of hIFN*α*-2b

The interferons (IFNs) were first introduced in 1957 as antiviral molecules. Based on their receptor types on the cell membrane surface, IFNs are classified into type I and type II. Type I consists of IFN*α*, IFN*β*, IFN*ω*, and IFN*τ*, while type II consists of IFN*γ*. IFN type I is a family of cytokines in which amino acid sequence similarity reaches 30–80%. Receptors that are recognized by the type I are grouped into two subunits, IFNAR-1 and IFNAR-2. IFNAR-2 consists of three types, namely, IFNAR-2a, IFNAR-2b, and IFNAR-2c, respectively. IFNAR-2c has important role in ligand binding and signal transduction, whereas IFNAR-2a and IFNAR-2b are competitive inhibitors that prevent IFN from binding to IFNAR-2c. IFN*α* has wide biological activities ranging as antiproliferation, immunomodulation, and antiviral. IFN*α* coding genes are located on human chromosome number 9. They have no introns and encode polypeptide chain of 165-166 amino acids. Some IFN*α*s are unglycosylated but some are glycosylated proteins with different degrees of glycosylation [7–9].

**Figure 1:**
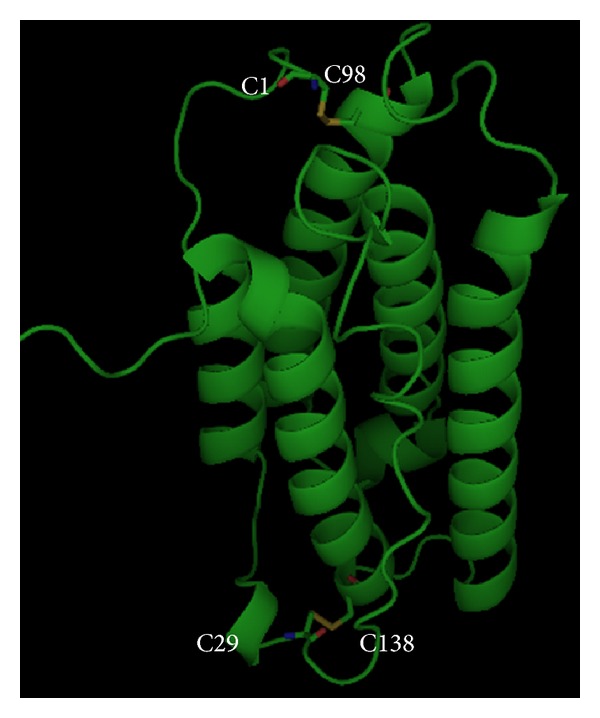
Molecular structure of hIFN*α*2b (by PyMOL).

hIFN*α*-2b molecule is a glycoprotein consisting of 166 amino acids with O-glycosylated threonine at position 106. Two disulfide bonds are formed by cysteines at position 1 and 98 as well as 29 and 138 (Figure 1). Bond formed by positions 1 and 98 is not required in biological activity. Amino acid residues that are important in the biological activity are Leu30, Lys31, Arg33, His34, Phe36, Arg120, Lys121, Gln124, Tyr122, Tyr129, Lys131, Glu132, Arg144, and Glu146. Based on crystal structure that is mediated by zinc dimer, each monomer of rhIFN*α*-2b consists of five alpha helices (called helix A to E) that are connected by a loop AB, BC, CD, and DE. Residues that are important in the structural integrity are Phe36, Tyr122, and Tyr129. Residues that are important in receptor binding are the AB loop (Arg22, Leu26, Phe27, Leu30, Lys31, Arg33, and His34), helix B (Ser68), helix C (Thr79, Lys83, Tyr85, and Tyr89), D helix (Arg120, lys121, Gln124, Lys131, and Glu132), and helix E (Arg144 and Glu146). Figure 1 shows a structure of rhIFN*α*-2b [4, 10, 11].

## 3. Molecular Mechanism of hIFN*α*-2b as Anticancer

Antiproliferative activity of hIFN-*α* consists of direct and indirect activities. Direct activity occurs through cancer cell growth inhibition by cell cycle arrest, apoptosis, or differentiation. Indirect activity occurs through activation of immune cells such as T cells and natural killer cells, inhibition of vascularization (antiangiogenesis), and induction of cytokines. The antiproliferative activity is the result of gene expression regulation. It is initiated by signal transduction pathways and transcriptional activation of JAK-STAT. Study of gene expression in melanoma cell lines (WM9), fibrosarcoma (HT1080), embryonic fibroblasts, and human dendritic cells reported that hIFN*α*s regulate more than 300 genes of signal transduction pathways in cells [8, 12].

**Figure 2:**
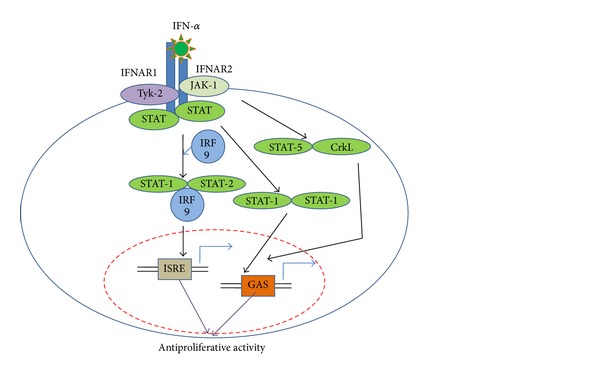
IFN*α*-2b signaling via JAK-STAT pathway, adapted from [13].

JAK-STAT pathway is initiated by receptors binding on the cell surface. JAK, a tyrosine kinase enzyme, can activate STAT through tyrosine phosphorylation (Figure 2). STAT family consists of seven proteins, namely, STAT-1, STAT-2, STAT-3, STAT-4, STAT-5a, STAT-5b, and STAT-6, respectively and JAK family consists of four proteins, namely, JAK-1, JAK-2, JAK-3, and tyrosine kinase-2 (TYK-2). JAK-1 and TYK-2 that are activated by IFN*α* will result in phosphorylation and dimerization. STAT protein-1 (P91) and STAT-2 (p113) subsequently translocated with interferon regulating factor-9 (IRF-9 or P48) to the cell nucleus. The protein complex known as IFN-stimulated gene factor 3 (ISGF-3) can activate interferon stimulating response element (ISRE). Two phosphorylated subunits of Stat 1 form alpha activation factor that binds to gamma activation sequence (GAS). These induce transcription of hundreds of interferon-stimulated genes (ISGs) that are involved in antiproliferative as well as antiviral activity [7, 8, 13, 14].

Mechanism of action of hIFN*α*2b in influencing the growth of various cancer cells occurs via the JAK-STAT signal transduction. JAK-STAT pathway related to the MAPK pathway as a major pathway in cell proliferation. MAPK pathway found in all eukaryotic cells and used to control a variety of processes in the cell, such as proliferation, differentiation, survival, and apoptosis. Proteins that play a role in this pathway are protein G and three protein kinases, namely, MAPK kinase kinase (MAPKKK), MAPK kinase (MAPKK), and MAPK. MAPKKK can phosphorylate and activate the MAPK protein kinase (MAPKK), and MAPKK may activate MAPK. hIFN*α*2b can inhibit extracellular signal-regulated kinase (ERK) mitogen ERK kinase (MEK) pathway, which includes a group of MAPK pathway. The pathway responds to growth factors and differentiation factors. MEK ERK pathway has Ras as protein G, Raf as MAPKKK, MEK as MAPKK, and ERK as MAPK. At the end of the signal transduction pathway, the transcription factor for mRNA genes synthesis that plays a role in the process of cell division is activated [15–17].

MEK ERK pathway inhibition by hIFN*α*2b has been widely reported. In experiments, using CD4 + T cells stimulated by anti-CD3 and interleukin-2 (IL-2), it was observed that hIFN*α*2b can prevent the G0/G1 phase of the cell cycle from entering S phase. As a result, cells cannot perform DNA replication and proliferation does not occur. Several publications reported that the inhibition of proliferation occurred because hIFN*α*2b may induce enzymes that can inactivate phosphatases PP2A and regulate docking protein that inhibits the interaction of ERK with MEK or MEK interaction with other kinases. Inhibition would decrease the activity of cyclin-dependent protein kinase (CDK-2 and CDK-4) and decreased the expression of cyclin D and E protein as the driving cell division. Inhibition of p21 expression leads to increase p27 Waf1/kip1 (inhibitor of cell division) and decrease phosphorylation of RB/p105 [18, 19].

**Figure 3:**
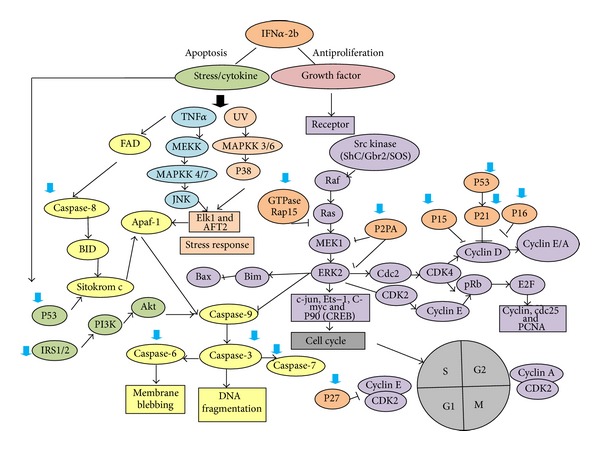
Molecular mechanism of hIFN*α*-2b as anticancer: apoptosis induction or cell cycle inhibition. The big arrow indicated overexpression of gene that is regulated by hIFN*α*-2b.

Mechanism of antiproliferation hIFN*α*2b occurs not only through regulation of protein synthesis and selective translation of proteins involved in inhibition of proliferation, but also through apoptosis as shown in Figure 3. There are two main pathways of apoptosis in cells that are activated: hIFN*α*2b family receptor signal transduction through tumor necrosis factor alpha (TNF-*α*) and the release of cytochrome c by mitochondria. Both of these pathways activate caspase signaling cascade resulting in DNA fragmentation and cell death. Induction of cell death occurs through TNF-*α* receptor family, namely, TNF-a/TNF-aR, FasL/Fas, Apo1, TRAIL/TRAILR, and Apo2. hIFN*α*2b contributes to an increase in p53 protein response to stress signaling and activation of p38 that plays a role in cell death. Additionally, hIFN*α*2b can activate PKR which has a variety of protein substrates such as eukaryotic initiation factor 2 (eIF2), NF-KB, IRF-1, p53, STAT1, and NF-90. These proteins may result in the control of cell division, differentiation, and apoptosis. PKR regulates transcription and translation to produce proteins Fas, p53, and Bax can trigger cell death via caspase pathway [14, 20, 21].

The expression of caspase pathway proteins, such as caspase-8 and caspase-9, caspase-3, caspase-6, and caspase-7, is also regulated by hIFN*α*2b. Caspase pathway is initiated by DNA damage signaling. The signal will cut BID protein and alter mitochondrial membrane permeability to release cytochrome c. Cytochrome c protein that activates Apaf-1 and caspase-9 will result in cell death. hIFN*α*2b also increases the protein expression of caspase-3 and caspase-7 as well as caspase-8 protein that produces DNA fragmentation. Stimulation of insulin receptor subunit (IRS1 and IRS2) activates phosphatidylinositol3-kinase (PI3K) as an inducer of apoptosis. The role of the PI3K/mTOR pathway in apoptosis remains unclear. PI3K has opposite function. It has been shown to function as a cell survival factor as well as an inducer of apoptosis. In tumor cells, PI3K/mTOR is necessary on apoptosis after treatment with IFN-*α*. The PI3K activation also leads to DNA fragmentation [14].

## 4. The Use of hIFN*α*-2b as Therapeutic Protein for Cancer Treatment

Interferons licensed for antitumor applications are hIFN-*α*2a (Roferon-A, Hoffmann-La Roche) and hIFN-*α*2b (Intron A, Schering-Plough). The most oncological indication of hIFN-*α*2b includes hairy cell leukemia, melanoma, follicular lymphoma, renal cell carcinoma, AIDS-related Kaposi's sarcoma, and chronic myelogenous leukemia. The clinical applications of rhIFN*α*-2b can be summarized in Table 1.

### 4.1. Hairy Cell Leukemia

Hairy cell leukemia is characterized by mononuclear cells of B-lymphocyte origin in the peripheral blood that have prominent cytoplasmic projections staining with tartrate-resistant acid phosphatase. It is also identified by its typical pattern of infiltration in the bone marrow and spleen. hIFN-*α*2b was first approved for use in hairy cell leukemia in 1986. The route of administration for hairy cell leukemia is subcutaneous and the recommended dose is 2 million U/m^2^ three times weekly for 12 months [22]. The first report of successful story of IFN was in 1984. Seven patients received 3 million U of partially purified (leukocyte) human IFN intramuscularly daily. Three of seven patients achieved a complete remission and four a partial remission [23]. Purified IFN*α*-2b synthesized by using recombinant DNA technology (Intron A, Schering Corporation) demonstrates similar activity [24]. It was also reported that hIFN*α*-2b was given to 50 patients. The dose was 2.0 × 10^6^ IU/m^2^ subcutaneously three times weekly. At 24 months, there were 38 patients remaining. During the two years of continuous IFN treatment none of the patients showed any signs of relapse. The IFN therapy was generally well tolerated, but 24 month evaluation showed mild toxicity in about 76% of the patients [25]. There was a study which reported unexpected high incidence of second neoplasm in patients after hIFN*α*-2b treatments with the same dose for 12 to 18 months of therapy. There were 13 patients from 69 patients (six were hematopoietic origin and the remaining were adenocarcinomas) who developed second neoplasm [26].

### 4.2. Melanoma

According to the national cancer institute, melanoma is defined as a form of cancer that begins in melanocytes. Melanocytes are cells that make the pigment melanin. Melanoma may begin in a mole (skin melanoma) but can also begin in other pigmented tissues, such as in the eye or in the intestines. The use of high dose of IFN2b for the adjuvant therapy of stage IIB and III melanoma patients was approved by FDA in 1995. A study by Kirkwood et al. [27] in 287 patients compared intravenous administration of hIFN*α*-2b at 20 MU/m^2^ for 1 month and subcutaneous administration at 10 MU/m^2^ for 48 weeks with observation alone. It was reported that prolongation of disease-free survival and prolongation of overall survival occurred in comparison to observation. In 1989, the Scottish melanoma group applied a randomized trial to compare observation alone with 6 months' therapy with subcutaneously low dose interferon at 3 MU/day (three times weekly). The result showed that there was a statistically significant improved disease-free survival for up to 24 months [28]. A Systematic Review of Randomized Controlled Trials by Lens and Dawes [29] stated that there was no clear benefit of hIFN*α*-2b on overall survival in melanoma patients. A large randomized controlled trial is needed to study the effectiveness and beneficiary of hIFN*α*-2b treatment.

### 4.3. Follicular Lymphoma

National cancer institute defines follicular lymphoma (FL) as a type of B-cell non-Hodgkin lymphoma that is usually indolent. The tumor cells grow as groups to form nodules. A meta-analysis to evaluate the role of hIFN*α*2 in FL reported prolongation of survival and remission duration when it has been given in the context of relatively intensive initial chemotherapy at 36 × 10^6^ units per month [30]. An East german study stated that rituximab added to first-line mitoxantrone, chlorambucil, and prednisolone chemotherapy followed by interferon maintenance prolongs survival in patients with advanced follicular lymphoma [31]. Another research reported that the role of interferon as initial and maintenance therapy in patients with newly diagnosed FL did not demonstrate any advantage. This study observed stage III or stage IV of 204 patients that receive either chlorambucil (CB), 10 mg daily for 6 weeks, followed by a 2-week interval, with 3 subsequent 2-week treatment periods at the same dose, separated by 2-week intervals, or CB given concurrently with interferon (IFN). IFN was given at a dose of 3 million units thrice weekly, subcutaneously, throughout the 18-week treatment period [32].

### 4.4. Renal Cell Carcinoma

The definition of renal cell carcinoma (RCC) according to the national cancer institute is a cancer that forms in the lining of very small tubes in the kidney that filter the blood and remove waste products. Long term results of combination of hIFN*α*2b and interleukin-2 administered subcutaneously in advanced renal cell carcinoma showed an improved survival for advanced RCC [33, 34]. Locatelli's study [33] stated that objective responses were observed in 9 of 50 (18%) patients and 6 of whom (12%) achieved a complete response. Overall median survival is 12 months; six patients were surviving at a median follow-up of 24 months, and three (6%) are still progression-free. It was also reported that nephrectomy followed by hIFN*α*2b administration improved survival of 120 metastatic RCC patients [35].

### 4.5. AIDS-Related Kaposi's Sarcoma

Kaposi's sarcoma is a form of skin cancer that can involve internal organs and mostly is found in acquired immunodeficiency syndrome patients. Treatment of Kaposi's sarcoma with hIFN*α*-2b in 114 patients using three dose regimens, that is, 50 × 10^6^ IU/m^2^ intravenously (high dose), 30 × 10^6^ IU/m^2^ subcutaneously (intermediate dose), or 1 × 10^6^ IU/m^2^ subcutaneously (low dose). Clinical responses were seen in all regimens and complete or partial remissions were obtained in 35% of the patients. In this study, it was known that high-dose therapy was associated with more rapid time to response [36]. Another study reported that low-dose IFN-*α*2b plus zidovudine seems to be a useful and well-tolerated therapy for KS with antitumoral and antiviral activity. This study observed the effect of combination of low dose of hIFN*α*-2b with zidovudine that administered 10–20 MU day^−1^ of hIFN*α* and 500–800 mg day^−1^ zidovudine in forty AIDS-associated Kaposi's sarcoma patients. Eighteen patients (45%) had an overall response (CR + PR) at 3 months and a response persisting for a median of 14 (3–27) months [37].

### 4.6. Chronic Myelogenous Leukemia

Chronic myelocytic leukemia (CML) is a cancer of white blood cells in which too many white blood cells are made in the bone marrow. The disease mostly characterized cytogenetically by the presence of the Philadelphia chromosome (Ph) (9;22)(q34;q11) in 90%–95% of patients [38]. A study of safety and efficacy of an indigenous rhIFN*α*-2b was conducted in 114 patients with chronic myelogenous leukaemia in India. All patients received 5 million units of rhIFN*α*-2b daily subcutaneously. The result confirmed that the protein efficacy with Kaplan-Meier probability of survival at 36 months was 76% [39]. Another study was applied in 82 Ph-positive CML patients that had intermittent or daily administration of rhIFN*α*-2b. The study reported the effectiveness of rhIFN*α*-2b in inducing clinical and cytogenetic response [38]. The rate of cytogenetic response and patients' survival increased when rhIFN*α*-2b was combined with cytarabine [40].

## 5. Conclusions and Future Directions

hIFN*α*-2b is a protein that has anticancer activity. It has been approved by the FDA as a therapeutic protein that can be used to treat some types of cancer, either in monotherapy or combination therapy with other anticancer drugs. The information regarding the side effects of rhIFN*α*-2b needs to be well known because its therapeutic use requires a long time. Development of rhIFN*α*-2b to improve the efficacy and safety of proteins is very important. The continuous further exploration on rhIFN*α*-2b will lead to the improvement of patient's life quality.

## Figures and Tables

**Table 1:** Summary of clinical application of rhIFN*α*-2b.

No	Indication	Clinical setting	Efficacy	References
1	Hairy cell leukemia	Seven patients received 3 million U of partially purified (leukocyte) human IFN intramuscularly daily.	Three of seven patients achieved a complete remission and four a partial remission.	[23]
		hIFN*α*-2b was given to 50 patients. The dose was 2.0 × 10^6^ IU/m^2^ subcutaneously three times weekly.	At 24 months, there were 38 patients remained. During the two years of continuous IFN treatment none of the patients showed any signs of relapse. The IFN therapy was generally well tolerated, but 24 month evaluation showed mild toxicity in about 76% of the patients.	[25]
		hIFN*α*-2b treatments with 2.0 × 10^6^ IU/m^2^ dose for 12 to 18 month therapy.	There were 13 patients from 69 patients (six were hematopoietic origin and the remaining were adenocarcinomas) developed second neoplasm.	[26]

2	Melanoma	Comparing intravenous administration of hIFN*α*-2b at 20 MU/m^2 ^for 1 month and subcutaneous administration at 10 MU/m^2^ for 48 weeks with observation alone in 287 patients.	Prolongation of disease free survival and prolongation of overall survival occurred in comparison to observation.	[27]
		A randomized trial to compare observation alone with 6 months' therapy with subcutaneously low dose interferon at 3 MU/day (three times weekly).	There was a statistically significant improved disease-free survival for up to 24 months.	[28]
		A Systematic Review of Randomized Controlled Trials by Lens and Dawes.	No clear benefit of hIFN*α*-2b on overall survival in melanoma patients. A large randomized controlled trial is needed to study the effectiveness and beneficiary of hIFN*α*-2b treatment.	[29]

3	Follicular Lymphoma	IFN*α*2b has given in the context of relatively intensive initial chemotherapy at 36 × 10^6^ units per month.	Prolongation of survival and remission duration.	[30]
		Rituximab added to first-line mitoxantrone, chlorambucil, and prednisolone chemotherapy followed by interferon maintenance.	Prolongs survival in patients with advanced follicular lymphoma.	[31]
		Stage III or stage IV of 204 patients that receive either chlorambucil (CB): 10 mg daily for 6 weeks, followed by a 2-week interval, with 3 subsequent 2-week treatment periods at the same dose, separated by 2-week intervals, or, CB given concurrently with interferon (IFN). IFN was given at a dose of 3 million units thrice weekly, subcutaneously, throughout the 18-week treatment period.	The role of interferon as initial and maintenance therapy in patients with newly diagnosed FL did not demonstrate any advantage.	[32]

4	Renal Cell Carcinoma	Long term of combination hIFN*α*-2b and interleukin-2 administered subcutaneously in 50 patients.	Objective responses was observed in 9 of 50 (18%) patients and 6 of whom (12%) achieved a complete response. Overall median survival is 12 months, six patients were surviving at a median follow-up of 24 months, and three (6%) are still progression-free.	[33]
		Nephrectomy followed by hIFN*α*-2b administration.	Improved survival of 120 metastatic RCC patients.	[35]

5	AIDS-related Kaposi's Sarcoma	Treatment of Kaposi's sarcoma with hIFN*α*-2b in 114 patients using three dose regimens, that is, 50 × 10^6^ IU/m^2^ intravenously (high dose), 30 × 10^6^ IU/m^2^ subcutaneously (intermediate dose), or 1 × 10^6^ IU/m^2^ subcutaneously (low dose).	Complete or partial remissions were obtained in 35% of the patients.	[36]
		Combination low dose of hIFN*α*-2b with zidovudine that administered 10–20 MU day^−1^of hIFN*α* and 500–800 mg day^−1^ zidovudin in fourty AIDS-associated Kaposi's sarcoma patients.	Eighteen patients (45%) had an overall response (CR + PR) at 3 months and a response persisting for a median of 14 (3–27) months.	[37]

6	Chronic Myelogenous Leukemia	114 patients with chronic myelogenous leukaemia in India. All patients were received 5 million unit of rhIFN*α*-2b daily subcutaneously.	Kaplan-Meier probability of survival at 36 months was 76%.	[39]
		82 Ph'positive CML patients that had intermittent or daily administration of rhIFN*α*-2b.	The rate of cytogenetic response and patients survival were increased when rhIFN*α*-2b was combined with cytarabine.	[40]
